# Compatibilization of Low Molecular Weight Polypropylene in High Molecular Weight Matrix Via Solvent Swelling

**DOI:** 10.1021/acsmacrolett.5c00732

**Published:** 2026-01-05

**Authors:** Carmen B. Dunn, Anthony Griffin, Smarika Neupane, Zhe Qiang

**Affiliations:** School of Polymer Science and Engineering, 5104 University of Southern Mississippi, 118 College Drive, Hattiesburg, Mississippi 39406, United States

## Abstract

Mechanical recycling of polypropylene (PP) causes chain scission during high-temperature, high-shear processing, resulting in lower molecular weight (MW) fragments, which contain fewer tie chains between crystalline lamellae. This change can lead to significantly reduced mechanical performance, making the use of post-consumer recycled PP in new materials particularly challenging, especially as recent government policies mandate the use of recycled content at increasing levels. Previous strategies in addressing these needs rely on chemically crosslinking or introducing additives, which can be time-consuming, cost-prohibitive at scale, and/or further complicate waste streams. In this work, we demonstrate a solvent immersion annealing method that swells PP blends containing high and low MW fractions, promoting their co-crystallization, which leads to significantly improved mechanical performance of the blended materials. Specifically, our results show an over 6-fold increase in extensibility of PP blends, effectively enabling their reuse and extended lifetime. This work presents an innovative and straightforward method to address the recycling challenges of low MW PP without the need for additives, potentially opening new avenues for future research in blend compatibilization for addressing plastic circularity challenges.

## Graphical abstract

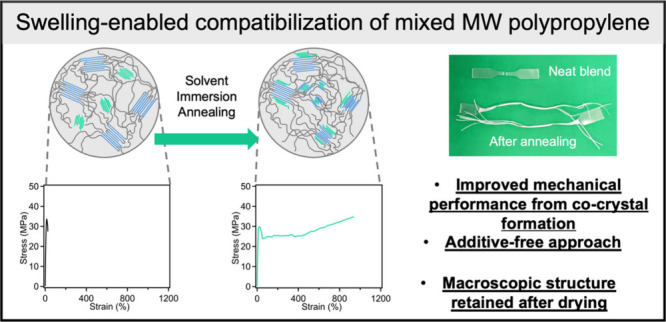

Enabling plastic circularity is crucial for achieving both environmental sustainability and long-term economic resilience in our society. However, polypropylene (PP), despite being one of the most widely produced plastics globally, has a very low recycling rate and presents a significant waste management challenge. In conventional mechanical recycling processes,^1,2^ the combination of oxygen exposure, high shear forces, and elevated temperatures often leads to hydrogen abstraction, initiating β-scission through the formation of stable tertiary radicals along the PP backbone.^3–6^ This results in significant chain scission and the generation of lower molecular weight (MW) PP fractions, which can negatively impact the mechanical properties of the recycled materials. Particularly, in recent years, various government policies have mandated the incorporation of post-consumer recycled (PCR) materials into new plastic production, with targets progressing from 20 to 50 wt % over the next decade.^7^ However, incorporating low MW PP (LPP), especially those with MW near or below the critical entanglement molecular weight (M_c_, ∼ 7000 Da),^8^ into virgin or commodity-grade PP, presents significant challenges even at low percentages. This is because these low MW PP chains lack sufficient tie chains between crystalline lamellae, leading to detrimental structural defects and poor mechanical performance.^9–11^ Several emerging strategies have been explored to enhance the performance of PP blends containing low MW fragments, including those relying on dynamic crosslinking chemistries to form reversible networks for improving mechanical properties.^12–15^ However, the introduction of crosslinking can hinder chain mobility and suppress polymer crystallinity, potentially compromising the desirable properties of virgin PP.^13,14,16^ As an alternative, non-reactive toughening agents, such as rubbers and inorganic fillers, can be used to improve the mechanical performance of blends containing low MW PP.^17,18^ While these approaches may enhance performance, they are often limited by costs and their introduction of additional components, which complicate the plastic waste stream and fundamentally reduce recyclability in future lifecycles. Therefore, developing an approach to improving the performance of PP blends containing low MW fractions, without relying on external additives, is both necessary and critical to overcoming key challenges in achieving the polyolefin circularity.

In this work, we demonstrate a solvent immersion annealing approach to effectively compatibilize low MW PP (average *M*_n_ ∼ 5000 g/mol, *M*_w_ ∼ 12 000 g/mol, Đ: ∼ 2.4; noted as LPP) into commodity-grade high MW PP (*M*_n_ ∼ 66 000 g/mol, *M*_w_ ∼ 420 000 g/mol, Đ: ∼ 6.4; noted as HPP), resulting in significant mechanical property improvement compared to neat blend samples. Specifically, we prepared a series of PP blends containing both high and low MW fractions with different ratio. Here, the LPP can represent degraded polymer chains typically formed during recycling processes, which exhibits significantly reduced extensibility and can lead to altered crystalline structures. Figure 1a shows the differential scanning calorimetry (DSC) thermogram of all virgin blends, while HPP exhibits a crystallinity degree of 36% and melting temperature (*T*_m_) of 161.8 °C. For LPP, two *T*_m_ values were observed, which may arise from stereodefects along the polymer backbone and/or differences in molecular weight within the sample.^19,20^ We note that LPP is very brittle and cannot even form a uniform sample after compression molding; the specimen cracks immediately upon cooling. This behavior is consistent with its low MW, which is below the critical threshold required to form sufficient tie chains for effective stress transfer (Figure S1a). The bimodal melting transitions were also observed in blend samples containing 5 wt % or more of LPP, with a second peak emerging at 145.3 °C as shown in Figure 1a; this secondary melting peak at a lower temperature corresponds to the presence of smaller crystal structures from LPP. As the LPP content increases to 20 wt %, the intensity of this secondary transition rises. The mechanical properties of these LPP/HPP blends are listed in Figure 1b. As anticipated, the incorporation of LPP into HPP can lead to inferior tensile performance compared to virgin HPP. At 5 wt % LPP, the blend can only withstand a 90 ± 15% tensile strain before failure and does not undergo any strain hardening. As LPP content is increased to 20 wt %, the strain at break further decreases, and the blend is unable to yield before fracture. Furthermore, with the introduction of 5 wt % LPP, the average toughness of sample substantially decreases from 450 ± 7 to 25 ± 7 MJ/m^3^; this decrease is more pronounced with higher fractions of LPP, and at 20 wt % LPP, we observe another drop in toughness to 7 ± 1 MJ/m^3^. Noteworthily, the modulus of blends does not change with the addition of 5 wt % LPP and remains between 450 and 550 MPa for all blend compositions.

**Figure 1:**
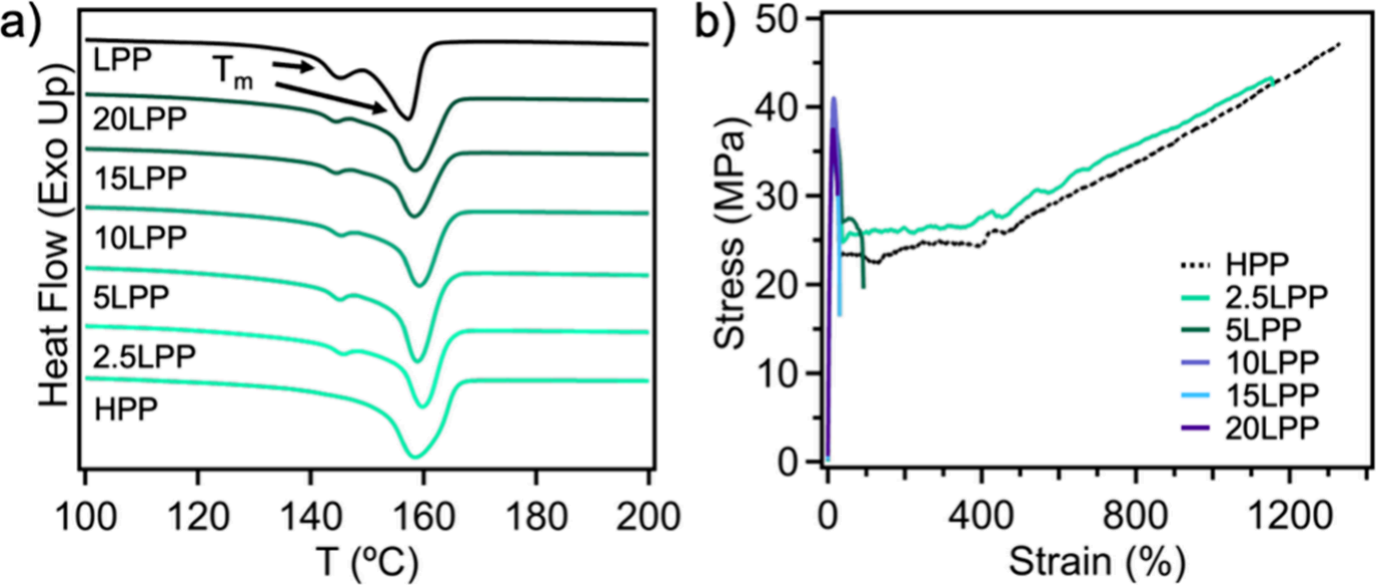
Presence of LPP from 2.5 to 20 wt % in HPP results in a) bimodal crystallites as shown in differential scanning calorimetry (DSC) heating scans and b) diminished mechanical performance and extensibility compared to neat HPP; brittle LPP fractures upon compression molding.

Our solvent immersion annealing method for treating these PP blends is simple and straightforward. Specifically, these PP blend samples were submerged in xylene at various temperatures ranging from 20 to 80 °C. Under these conditions, the samples remained translucent, and their macrostructure exhibited only a moderate degree of isotropic swelling (Figure S1b). Specifically, at 20 °C, the dimensional change of 5LPP samples reached 4.0 ± 0.1%. As the temperature increased to 40 °C, 60 °C, and 80 °C, the degree of swelling increased to 4.3 ± 0.2%, 5.0 ± 0.5%, and 6.2 ± 0.2%, respectively, indicating elevated temperatures promote sample expansion (Figure 2a). When the immersion annealing temperature exceeded 90 °C, the samples became significantly distorted, indicating excessive solvent uptake and structural instability. Furthermore, the sample swelling kinetics of 10LPP is shown in Figure S1c, demonstrating an equilibrium swelling time of 8 h at 60 °C. To further examine the crystal structures of the LPP and HPP during the immersion annealing, both small- and wide-angle X-ray scattering (SAXS and WAXS) were performed before, during, and after solvent immersion annealing at different temperatures. *In-situ* SAXS profiles shown in Figure 2 (b–c) exhibit a primary ordering peak across all samples, indicating that both LPP and HPP remain semicrystalline during solvent annealing. This phenomenon indicates that the solvent molecules primarily penetrate in and swell the amorphous domains, leaving a limited impact on the crystalline regions. As shown in Figure 2d, the long period (*d*), containing both the crystalline and amorphous regions of the PP, was calculated with the following relation

$$d=\frac{2\pi }{q^{*}}$$ 1

where q* corresponds to the position of primary scattering peak. For neat LPP, solvent immersion annealing increases *d* from 11.0 nm in unannealed samples to 12.1 nm with the introduction of xylenes. At elevated temperatures of 50 °C, 70 °C, and 90 °C, *d* further increases to 12.7, 13.3, and 13.9 nm, which correspond to a swelling degree of 15%, 21%, and 26%, respectively (Figure 2d). For HPP, the initial long period of 12.8 nm increases further to 14.3 nm at 60 °C, corresponding to an ∼ 11% swelling degree. Notably, at the same temperature, the measured increase in sample dimensions (macroscopic scale, ∼ cm; Figure 2a) is lower than the long-period expansion degree of PP crystals (mesoscale, ∼ nm, as determined by *in situ* SAXS, Figure 2d). This discrepancy is likely due to the fact that dimension measurements of samples were taken immediately after removal from the solvent bath, during which partial shrinkage from solvent evaporation may have already occurred. An increased peak area of the primary scattering peak was observed in LPP (Figure 2b), indicating a loss of ordering in the crystal structures in xylene with increased temperature. WAXS plots of neat and annealed LPP and HPP can be found in Figure S2a, indicating that the crystal structure is retained after solvent immersion annealing and drying. Furthermore, in situ WAXS results of LPP (Figure S2b) and HPP (Figure S2c) in xylene, which show characteristic peaks at (110), (040), (130), and (111), suggest retained α-crystal structure during the solvent immersion annealing process.^21,22^ However, the full width at half-maximum (FWHM) of the (110) peak of LPP samples increases from 0.80 nm^–1^ at 30 °C to 0.87 nm^–1^ at 90 °C annealing, which indicates a higher temperature can cause further disruption in their crystal structures. In constrast, for HPP materials, the same (110) peak maintains a FWHM of ∼ 1.0 nm^–1^ between 30 and 90 °C, indicating no significant change to its crystallinity. Upon drying, the primary ordering peak observed in SAXS is similar to neat blend samples, indicating that solvent removal led to the recovery of the original domain spacing, as shown in Figure S3.

**Figure 2:**
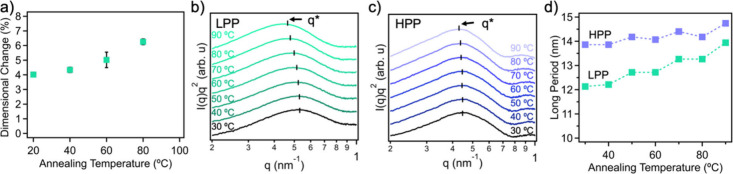
a) Dimensional change of 5LPP blends when immersed in xylene at different temperatures, Lorentz-corrected in situ SAXS curves of b) LPP and c) HPP in xylenes at different temperatures, d) long period of solvent immersion annealed HPP and LPP at different annealing temperatures.

The LPP/HPP blends at various ratios were annealed in xylenes at 60 °C for 24 h, and their mechanical properties were subsequently characterized. As shown in Figure 3b, the 2.5LPP tensile bars reached strains of up to 1250% before failure, which is 1.25 times higher than neat blend prior to annealing and comparable to HPP. Likewise, 5LPP blends were extensible up to 1027 ± 98% and retained toughness of 313 ± 43 MJ/m^3^. In fact, blends of up to 10 wt % LPP undergo yielding and strain hardening and are extensible up to 975 ± 90% before failure. Importantly, the modulus of annealed blends of LPP/HPP is retained between 440 ± 5 (2.5LPP) and 507 ± 34 MPa (15LPP). This strong mechanical performance is commensurate with some previous reports, which employ copolymer additives to induce cocrystallization.^23–27^ Furthermore, mechanical properties of HPP alone show comparable mechanical performance to their untreated analog, showing toughness of 425 ± 41 MJ/m^3^ and strain at break values of 1350 ± 96%, suggesting the mesoscale structural rearrangement of LPP/HPP blends provide a key mechanism to promote their performance improvement (Figure S4a). A gradual decrease in blend toughness was observed in the annealed blend samples as the LPP content increased, indicating that our method may become less effective with a higher presence of defective sites. This trend is further confirmed in the 20LPP sample, which remained brittle even after annealing, indicating that the extent of the low MW content can outweigh the restorative effects enabled by solvent immersion annealing. It is important to note that our method is fundamentally distinct from other recent works typically employing additives or crosslinkers to impart enhanced mechanical performance on fragile polyolefins; these methods led to a reduced elastic moduli compared to virgin PP (due to reduced crystallinity), reaching approximately 140 MPa.^13^ Furthermore, even dynamic cross-linking strategies for upcycling low MW polyolefins result in the compromise of extensibility, which was not observed for our immersion annealing process.^28^ Our strategy for improving the mechanical performance of compatibilized blends is also distinct from previous efforts in PP composition/formulation design, which typically relies on incorporating polymers with broad molecular weight dispersity to enhance processability and toughness;^29–31^ in our case, solvent annealing enables engineering of polyolefins in their semicrystalline state to mitigate defects (e.g., lack of tie chains) that were present in their neat state, promoting extensibility and toughness. Importantly, the *T*_g_ of our annealed LPP/HPP blends increases following solvent immersion annealing and complete solvent removal (Figure S5), and thermogravimetric analysis of dried samples revealed the absence of residual solvent (Figure S6), indicating that the enhanced sample extensibility is not due to plasticization effect. Instead, the annealing process appears to promote chain ordering, leading to a slight increase in the *T*_g_.

**Figure 3:**
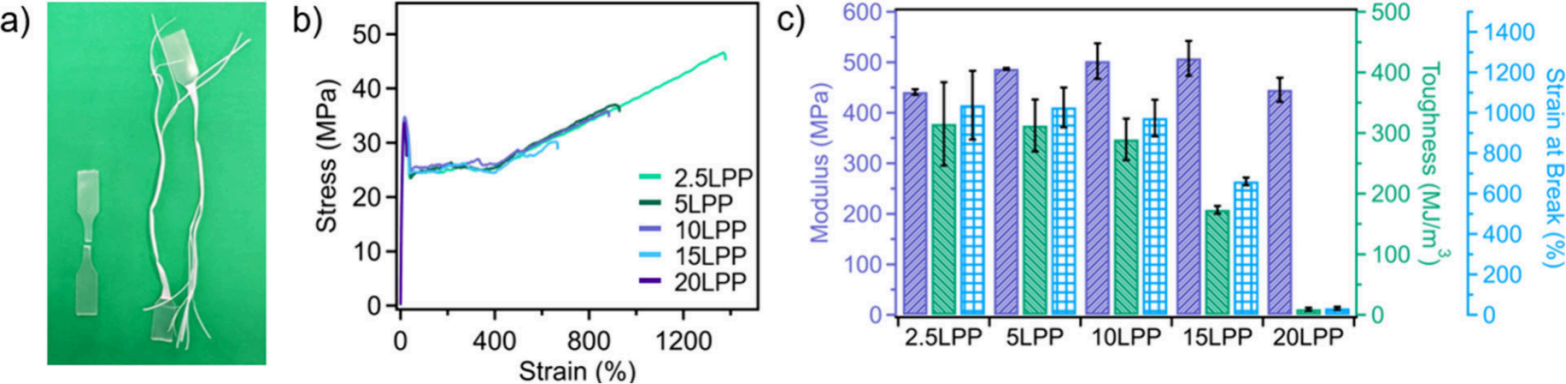
a) 10LPP extensibility before (left) and after (right) solvent immersion annealing at 60 °C for 24 h, b) stress–strain curves of 2.5LPP, 5LPP, 10LPP, 15LPP, and 20LPP after solvent immersion annealing at 60 °C for 24 h, demonstrating improved mechanical performance, and c) the modulus, toughness, and strain at break values for LPP/HPP blends after solvent immersion annealing under the same conditions.

To further understand the origins of the improved mechanical performance in LPP/HPP blends following immersion annealing, differential scanning calorimetry (DSC) was conducted to characterize the crystalline structures of the respective components. Figure 4a presents the first heating cycles of the annealed LPP/HPP blends, which all exhibited a single melting transition temperature (*T*_m_) in contrast to the bimodal melting observed in unannealed blends. The absence of a lower *T*_m_ from LPP, combined with SAXS results indicating retained semicrystalline structures during annealing, suggests that the LPP can be co-crystallized within the HPP matrix from our process. Full thermograms for both unannealed and annealed LPP/HPP blends are listed in Figure S7, while crystallization temperatures (*T*_c_), extracted from the cooling curves, are shown in Figure 4b. These results reveal a consistent increase in *T*_c_ across all blends after solvent immersion annealing, along with a shift toward higher *T*_m_ values at elevated LPP concentrations (Figure S8).^32^ When comparing annealed and neat HPP, there is an apparent decrease in the degree of crystallinity (*X*_c_) of 4% (Figures S8a and S4b). We attribute this behavior to the fact that during the immersion step a portion of the PP crystallites melt becomes amorphous. Upon solvent removal and sample cooling, these chain segments do not have sufficient mobility to refold into their original ordered state, thereby reducing the overall crystallinity, which also explains why the *T*_m_ peak of HPP from the DSC scan became broader. However, when LPP was included in the blend, we observed an increase in *X*_c_ of annealed samples with higher LPP content, up to 15LPP, at which point the neat and annealed samples show comparable *X*_c_ values. Furthermore, the lamellar thickness (L) of these PP crystals was calculated with the Gibbs–Thomson equation,

$$T_{m}=T_{m}^{0}\left(1-\frac{2\sigma_{e}}{\Delta H_{f}^{0}L}\right)$$ 2

where *T*_*m*_^0^ is the equilibrium melting temperature of the crystalline lamella (*T*_*m*_^0^ = 481 K), *T*_*m*_ is the measured melting temperature via DSC, Δ*H*_*f*_^0^ is the enthalpy of fusion of the crystalline phase of PP (Δ*H*_*f*_^0^ = 207 J/g), and σ_*e*_ is the surface energy of the basal surface of a lamellar crystal (σ_*e*_ = 62.3 × 10^–7^ J/cm^2^).^33,34^ The calculated values of *L* for annealed and unannealed PP blends are listed in Figure 4c. Upon the addition of 2.5 wt % LPP to HPP, there is a reduction in L from 6.2 to 5.9 nm due to the presence of imperfect LPP structures disrupting the crystal spherulites, making them smaller. Upon solvent immersion annealing, *L* is slightly reduced to 5.7 nm in 2.5LP. We note previous literature results show that smaller and more uniform crystallites within semicrystalline polymers can improve their ductility.^35–37^ As the LPP content increases, the reduction in *L* becomes less pronounced following immersion annealing, suggesting a limited ability of this method to reorganize the crystalline structure of blends with a higher LPP content in xylene at 60 °C. Moreover, polarized optical microscopy (POM) was performed on 5LPP before, during, and after annealing. The images can be found in Figure S9 and depict comparable size distributions of spherulites before and after immersion annealing.

**Figure 4:**
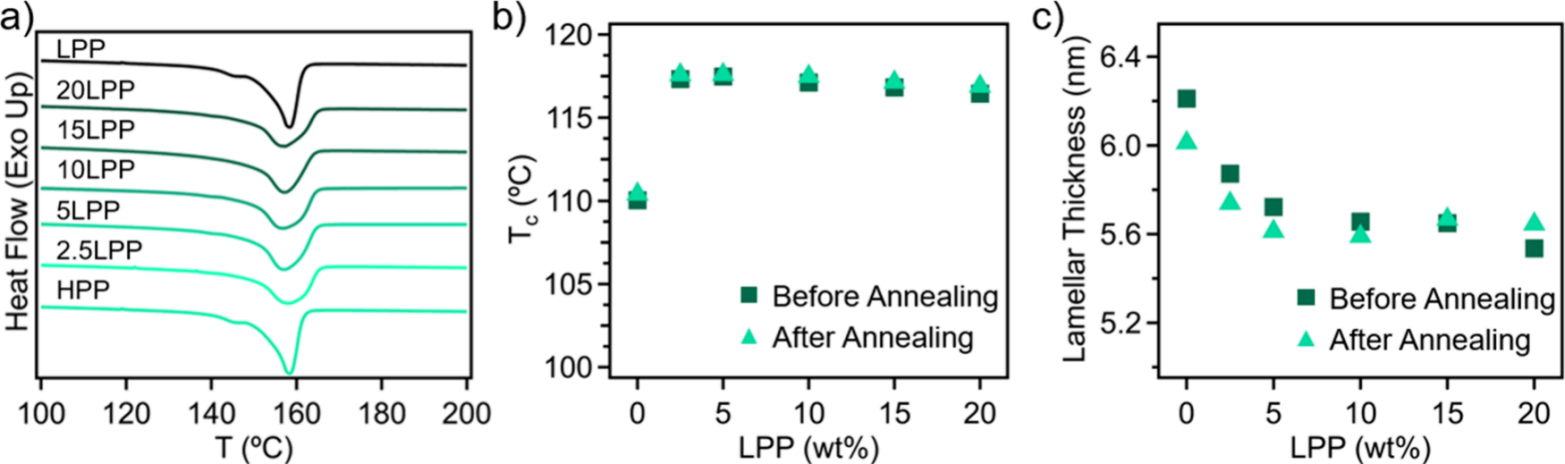
a) Monomodal melting transitions of LPP/HPP blends were observed from the first heat of DSC heat–cool-heat thermograms, b) the peak crystallization temperature for annealed and unannealed LPP/HPP blends taken from the cooling curves of DSC thermograms, and c) the lamellar thickness of LPP/HPP blends calculated from the *T*_m_ (neat LPP = 5.7 nm, annealed LPP = 5.8 nm).

Building on these experimental observations, Figure 5 illustrates the mechanistic concept of the solvent immersion annealing method to compatibilize HPP and LPP via cocrystallization. Specifically, it is found that solvent immersion annealing at controlled temperatures can swell the amorphous domains of HPP and LPP while retaining their semicrystalline structure, enabling improved intermolecular chain entanglement, facilitating cocrystallization behaviors, and tie-chain trapping. We note that this process is effective and can be scalable because the amorphous domains are continuous in nature, allowing solvent diffusion throughout the entire sample without being limited by sample dimensions. Additionally, the retention of crystalline order in these blends during annealing supports their macrostructural stability. Upon removal from the xylene, these samples can fully recover to their original shape and dimension. Therefore, this process is applicable to parts post manufacturing, resulting in reduced defect sites, particularly those related to the absence of tie chains between lamellae, which in turn can lead to significantly enhanced mechanical properties in the final PP blends. We have also demonstrated that the cocrystal formation induced by immersion annealing is a kinetically driven process. After subjecting these samples to an additional thermal annealing step to melt and recrystallize, the blend becomes brittle again (Figure S10). This behavior is consistent with the re-appearance of two distinct melting peaks in Figure S7, where the second heating scan after erasing the prior thermal/annealing history (green curve) shows two separate melting transitions corresponding to the individual PP crystals, similar to their initial state after compounding.

**Figure 5:**
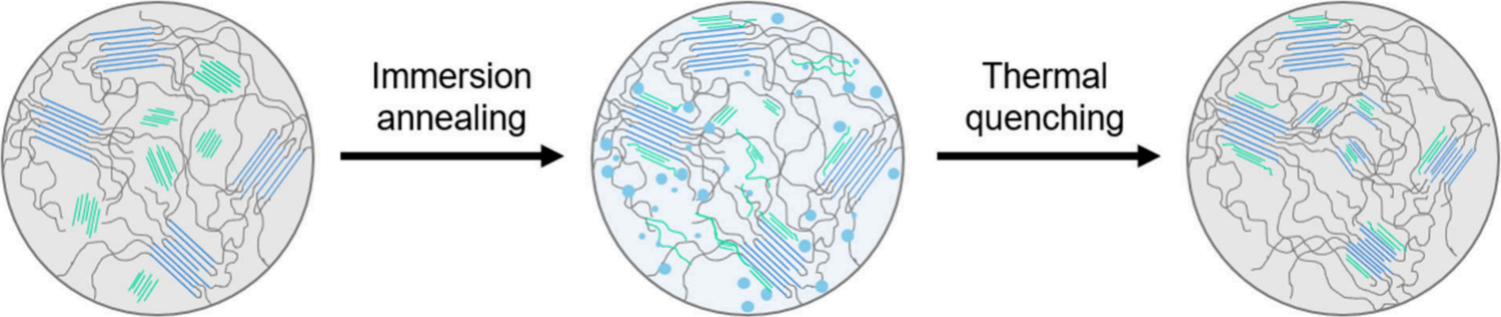
Through solvent immersion annealing, the amorphous domains of the HPP expand, enabling LPP to both cocrystallize with HPP and entangle with its amorphous region. These cocrystals are retained upon thermal quenching.

Although the improvement for the 20 wt % LPP sample was very limited from immersion annealing at 60 °C, increasing the annealing temperature to 80 °C led to a marked enhancement. Specifically, Figure 6 illustrates the stress–strain curves of 20LPP in tension after annealing at 60 and 80 °C for 8 h, respectively. We observe the degree of swelling and annealing temperature can have a significant impact on blend performance. At the annealing temperature of 60 °C, we observe a toughness of only 9.3 ± 2.4 MJ/m^3^ and a strain at break of only 33 ± 7% for 20LPP, suggesting the limited interactions between HPP and LPP (i.e., cocrystallization). However, increasing the annealing temperature to 80 °C drives enhanced swelling of HPP amorphous domains, enabling the disruption of a larger number of LPP crystals. The resulting material after annealing and drying is able to withstand a strain of 814 ± 127%. This result indicates that a higher temperature, which enhances the degree of swelling, can further improve the mechanical properties of blends containing larger quantities of LPP.

**Figure 6:**
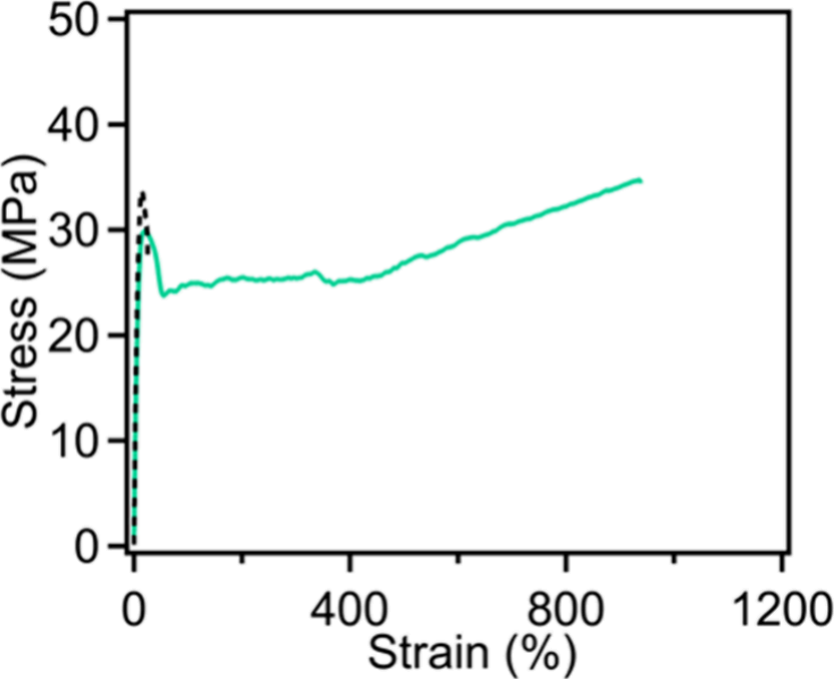
Tensile curves of 20LPP annealed at 80 °C (solid/green) and 60 °C (dashed/black).

In summary, we present a simple and robust method for compatibilizing LPP within an HPP matrix, resulting in significantly enhanced mechanical performance compared to untreated blends. This process relies on solvent immersion annealing, which selectively swells the amorphous regions of the polymer. Compatibilization is achieved through a combination of cocrystallization between HPP and LPP and the formation of kinetically trapped molecular entanglements. Although solvent annealing could perturb the crystalline structure, both HPP and LPP remain semicrystalline in solvent up to 90 °C. This strategy can be applied to manufactured parts for enhancing their mechanical performance without the need for reactive groups or compatibilizer additives, offering a promising route for process-driven upcycling of polyolefins.

## Supplementary Material



## Notes

Experimental section containing materials list, protocol for sample preparation, and materials characterization; images of 5LPP tensile bars before and after immersion annealing and immersion annealing swelling kinetics of 10LPP at 60 °C; WAXS plots of neat and annealed HPP and LPP, and LPP and HPP in xylenes at increasing temperature; tensile curves and melting transitions of neat and annealed HPP; thermogravimetric analysis data for annealed HPP/LPP blends; differential scanning calorimetry cooling thermograms before and after annealing of HPP/LPP blends and their measured glass transition temperatures; differential scanning calorimetry heat–cool-heat thermograms of neat and annealed HPP/LPP blends; degree of crystallinity and peak melting temperature as functions of LPP concentration and solvent annealing; and polarized optical microscopy images of 5LPP spherulites before, during, and after solvent annealing (PDF)
