# Supplementary material for: Compatibilization of Low Molecular Weight Polypropylene in High Molecular Weight Matrix Via Solvent Swelling
Source: ACS Macro Lett. 2026 Jan 5;15(1):189–95. doi: 10.1021/acsmacrolett.5c00732 (PMC12825374; doi:10.1021/acsmacrolett.5c00732)
Supplement: Supplementary file 1 [file mz5c00732_si_001.pdf]

## Supporting Information For:

**Compatibilization of low molecular weight polypropylene in high molecular weight matrix via solvent swelling**

Carmen B. Dunn[1], Anthony Griffin[1], Smarika Neupane[1], Zhe Qiang[1*]

[1] School of Polymer Science and Engineering, University of Southern Mississippi, *118 College Drive, Hattiesburg, Mississippi 39406, United States*

[*]Correspondence to Z. Q. (Email: zhe.qiang@usm.edu)

## Experimental Section

### Materials

Low molecular weight polypropylene (isotactic, average $M_n$ ~ 5,000 g/mol, $M_w$ ~ 12,000 g/mol, Đ: ~2.4; noted as LPP) was obtained from Sigma-Aldrich. High molecular weight polypropylene (extrusion-grade, high molecular weight commodity plastic, average $M_n$ ~ 66,000 g/mol, $M_w$ ~ 420,000 g/mol, Đ: ~6.4; noted as HPP) was obtained from Muehlstein. Xylenes (99%) was obtained from Thermo Fisher Scientific.

### Sample Preparation

All formulations were compounded using an Xplore MC5 microcompounder at 190 ºC and a screw speed of 100 rpm. Blends are named with the convention of (weight percent LPP) LPP. For an example blend of 5LPP, pellets of HPP (2.85 g) and LPP (0.15 g) were pre-mixed and then introduced into the microcompounder. The residence time for each batch was held at 5 min before extrusion. Materials were then distributed into rectangular aluminum molds and compression molded with a Carver press at 5000 psi for 5 min at 190 ºC before cooling between two metal plates until samples reached room temperature. Samples were then punch-pressed into Type V tensile bars (w = 3.25 mm, t = 0.75 mm, l = 12.25 mm) for further study.

In a typical solvent immersion annealing protocol, the width and thickness of each tensile bar's gauge was measured with calipers, and the mass of each tensile bar after swelling was recorded. Samples were submerged in xylenes and held at a temperature between 20 – 100 ºC for a time between 2 and 24 h. Upon removal from xylenes, each bar was wiped dry, and the dimensions of the gauge were measured again. Each tensile bar was allowed to dry at room temperature for 2 d before vacuum drying at room temperature to remove all residual xylenes for 24 h. Upon drying, final sample dimensions were measured and mass was recorded.

### Materials Characterization

Tensile tests of annealed and control samples were performed on a Mark-10 EasyMESUR motorized test stand with wedge grips and a 250 N load cell. The extension rate was held at 15 mm/min at room temperature, based on ASTM 638 with a type V tensile bar. Data analysis was performed on Igor Pro 9, where the elastic modulus was calculated from the linear region prior to sample yielding, and the toughness was determined through integration of the stress-strain curves for each sample. The melting, crystallization, and glass transition temperatures ($T_m$, $T_c$, and $T_g$) of samples before and after solvent immersion annealing were determined using differential scanning calorimetry (DSC) with a TA Instruments Discovery DSC250. Tzero pans and lids from TA Instruments were used, and a heat-cool-heat cycle was employed with a temperature profile of -90 to 220 ºC with a ramp rate of 10 ºC/min to identify $T_c$ and $T_m$ from the second and third step respectively. The degree of crystallinity ($X_c$) of blends before and after annealing was calculated by integrating the crystalline melting transition peak (between 140 °C and 165 °C), which was taken as $\Delta H_f$. Then, $X_c$ was calculated as follows

$$X_c = \frac{\Delta H_f}{\Delta H_f^0} \times 100\%$$

where $\Delta H_f^0$ is the reference enthalpy of 207 J/g for a perfect PP crystal. In a separate ramp of DSC to -90 ºC, the midpoint of the step transition was taken as $T_g$. These specific cooling ramps were used to obtain the $T_g$ directly after annealing without removal of thermal history.

Thermogravimetric analysis (TGA) was conducted on annealed and dried PP blends at a ramp rate of 10 °C/min under $N_2$ up to 600 °C. Mass change was also recorded for blends by measuring with a tared analytical balance before annealing and after drying.

Both small-angle and wide-angle X-ray scattering (SAXS and WAXS) were performed on fibral and powder samples of HPP and LPP using a Xeuss 3.0 system (Xenox, Grenoble, France) equipped with a GeniX beam delivery system, providing monochromatic Cu and Kα radiation. Each sample was loaded into a 1.5 mm capillary and subsequently filled with xylenes; the capillary was then loaded into a temperature-controllable Linkam stage, and each measurement was taken one minute after the stage reached the desired temperature. Scattering patterns were collected using a Dectris Eiger 2R 1M-pixel 2D detector and reduced into an integrated 1D profile. The sample distances for all SAXS and WAXS measurements were 900 mm and 45 mm respectively. The samples were exposed to the X-ray beam for 300 s (WAXS) or 600 s (SAXS) for measurement. Measurements were taken at 30, 40, 50, 60, 70, 80, and 90 $^0$C for each sample. Additional profiles were collected of pre-annealed and neat samples with the same experimental parameters at room temperature using a solid sample stage.

Polarized optical microscopy (POM) (OLYMPUS BX53M) was conducted with a temperature controller (Instec mK100, HCS302). Samples of neat and pre-annealed 5LPP were imaged at room temperature, and in-situ annealed 5LPP was imaged at 60 and 80 $^0$C using the same optical setup. All polarized images were taken on a Q-Imaging RoHS Camera (2052 pixel x 1086 pixel) in a transmission mode at a 90 $^0$ angle configuration between the linear polarizer and the linear analyzer.

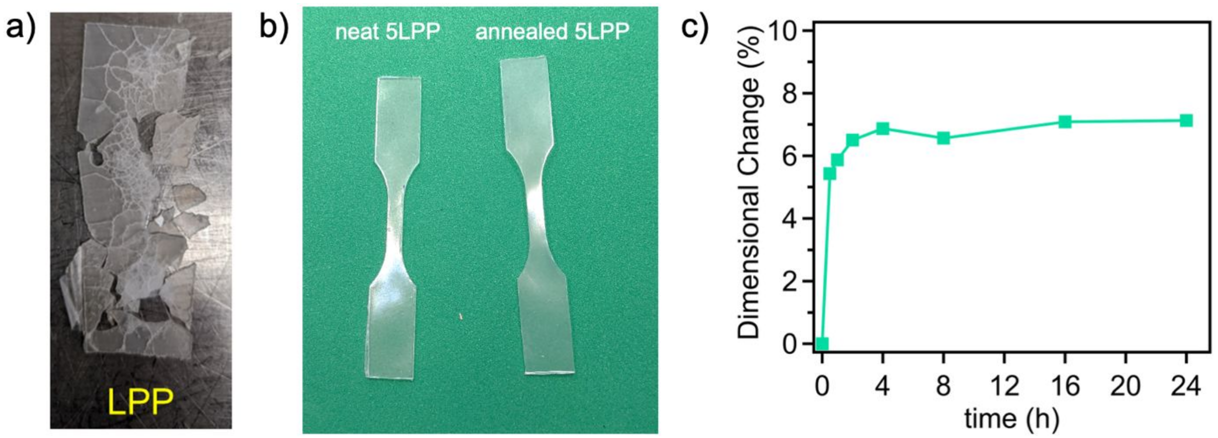


**Figure S1**. a) photo of a LPP specimen which cracks immediately after cooling in the compression mold, b) 5LPP tensile bars before (left) and immediately after (right) immersion annealing at 65 $^{0}$C for 24 h (in swollen state), and c) swelling kinetics of 10LPP at 60 $^{0}$C demonstrating dimensional change as a function of annealing time.

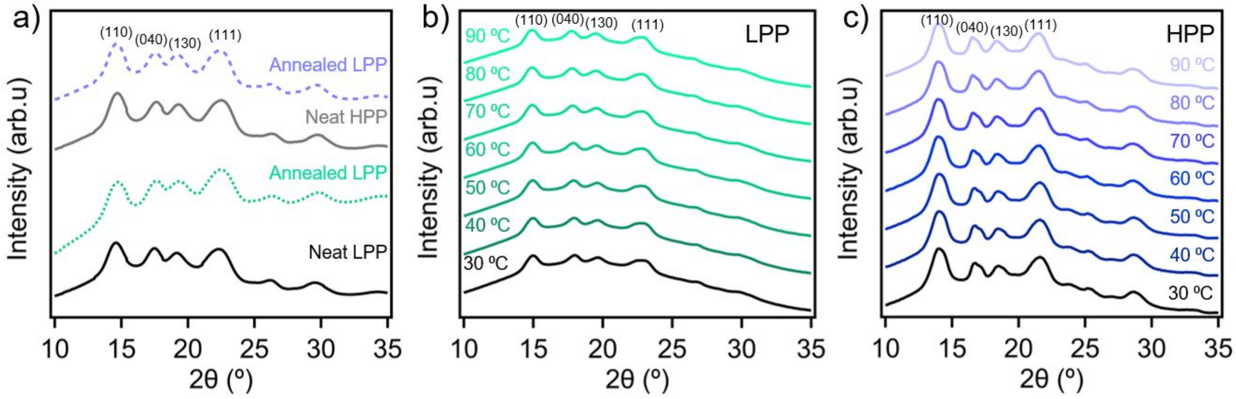


**Figure S2**. WAXS plots of a) neat and annealed HPP and LPP, b) LPP in xylenes at increasing temperature, and c) HPP in xylenes at increasing temperature.

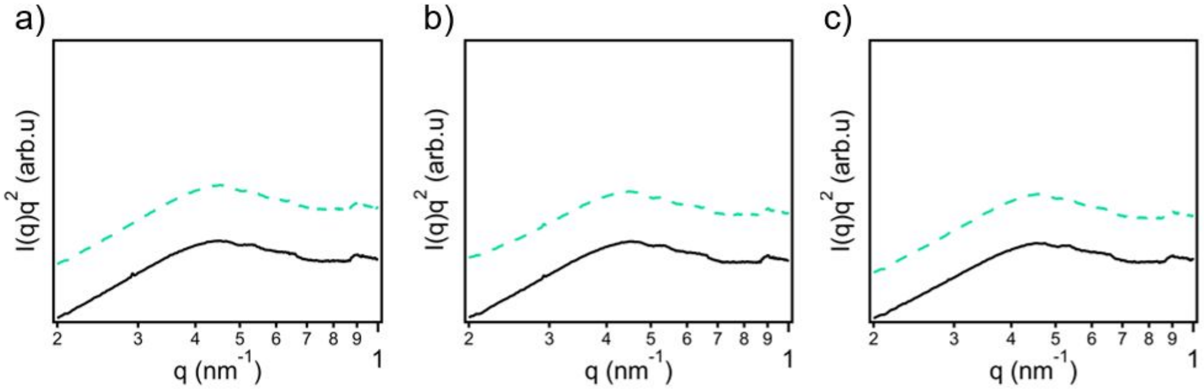


**Figure S3**. Lorentz corrected SAXS profiles before (solid/black) and after (green/dashed) immersion annealing of a) 5LPP, b) 10LPP, and c) 20LPP.

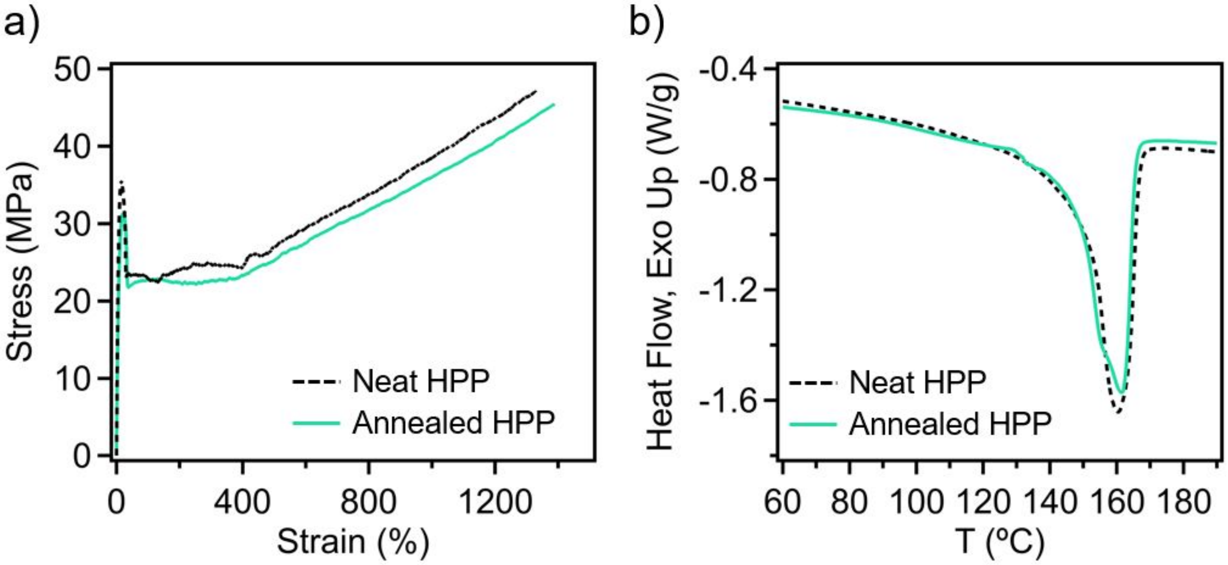


**Figure S4.** a) Tensile curves of neat and annealed (60 $^0$C xylenes, 24 h) HPP and b) melting transitions taken from DSC thermograms of neat and annealed (60 $^0$C xylenes, 24 h) HPP.

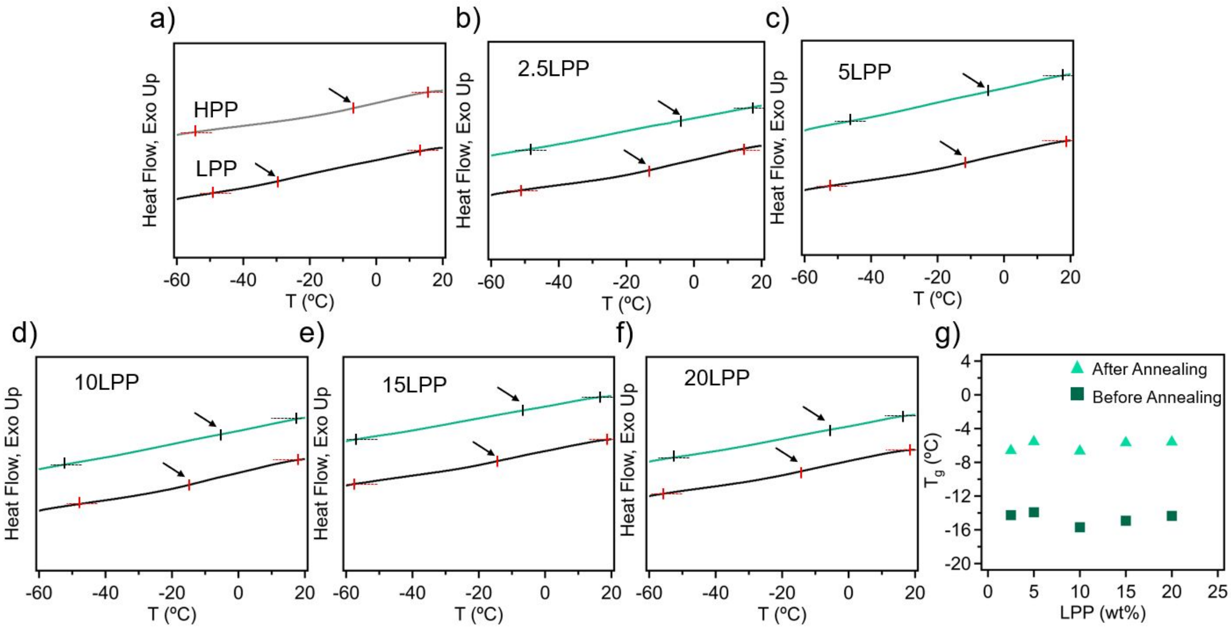


**Figure S5.** Differential scanning calorimetry cooling thermograms before annealing (black) of a) HPP and LPP and before (gray/black) and after (green) annealing of b) 2.5LPP, c) 5LPP, d) 10LPP, e) 15LPP, and f) 20LPP, where g) glass transition temperatures were taken from the midpoint of each step transition.

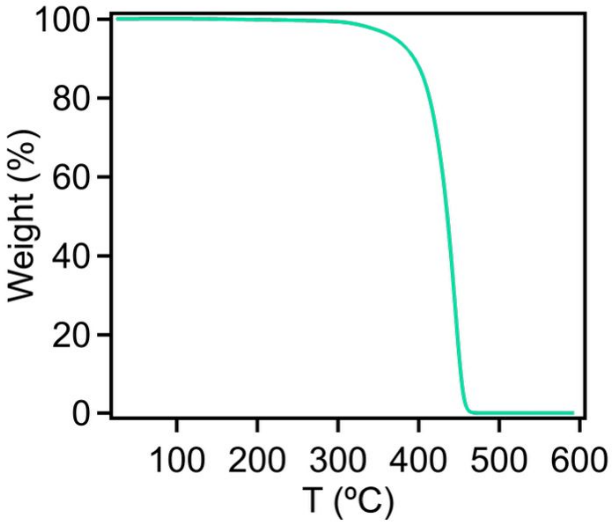


**Figure S6.** TGA of dried 20LPP after annealing at 60 °C

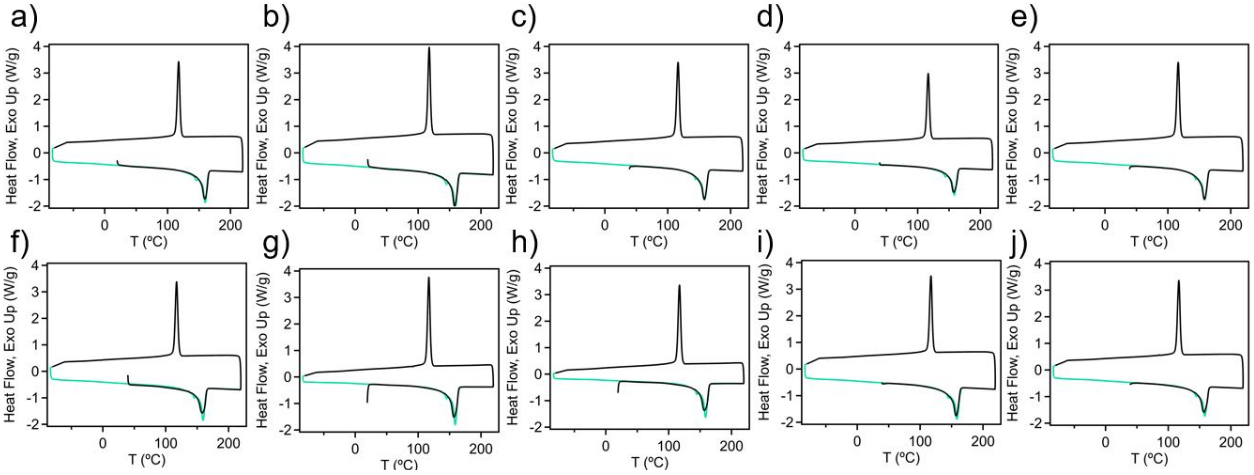


**Figure S7.** Differential scanning calorimetry heat-cool-heat thermograms of neat samples of a) 2.5LPP, b) 5LPP, c) 10LPP, d) 15LPP, e) 20LPP, and annealed samples of f) 2.5LPP, g) 5LPP, h) 10LPP, i) 15LPP, and j) 20LPP.

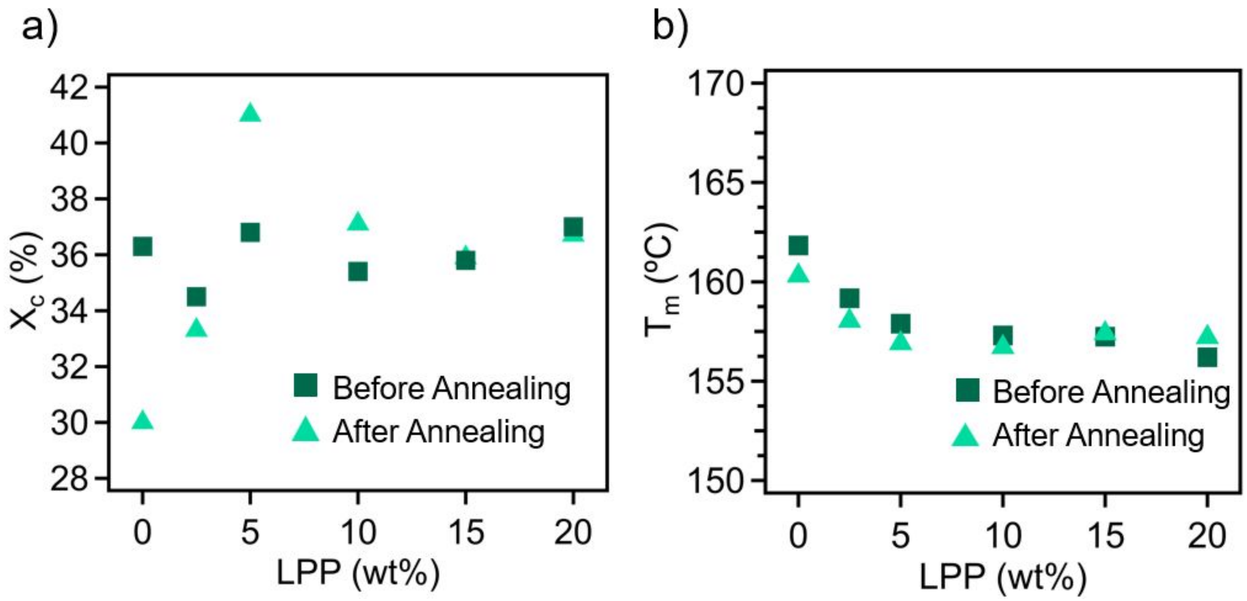


**Figure S8.** a) the degree of crystallinity and b) the peak melting temperature as functions of LPP concentration and solvent annealing.

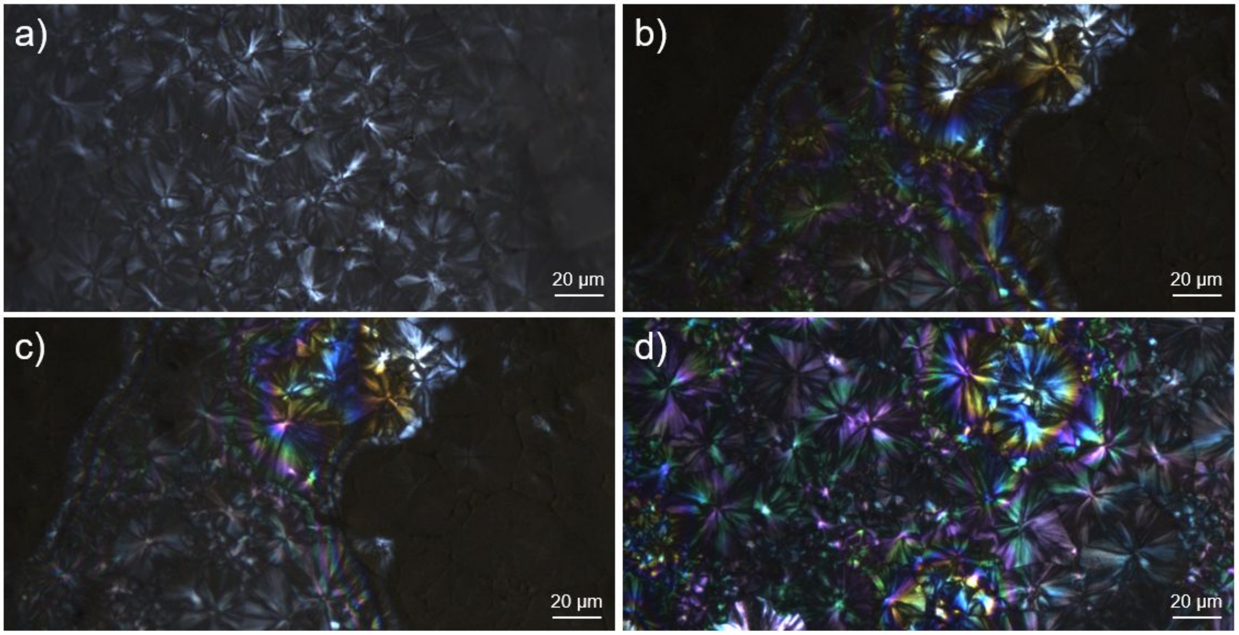


**Figure S9.** Polarized optical microscopy (POM) images taken at 50x of 5LPP spherulites a) before annealing, b) during solvent annealing at 60 $^0$C, c) during solvent annealing at 80 $^0$C, and d) after solvent annealing at 60 $^0$C and drying.

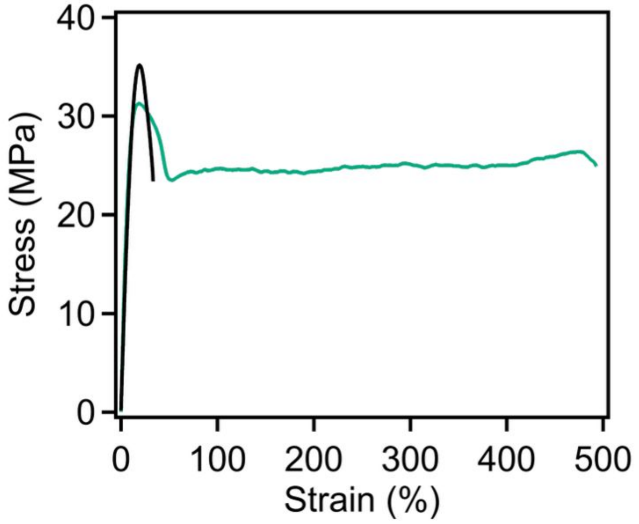


**Figure S10.** Stress-strain curves of 15LPP after solvent annealing (green) and after solvent annealing and an extra step thermal annealing at 160 $^{0}$C for 5 min (black).
